# From Standard to Escalated Anticoagulant Prophylaxis in Fractured Older Adults With SARS-CoV-2 Undergoing Accelerated Orthopedic Surgery

**DOI:** 10.3389/fmed.2020.566770

**Published:** 2020-10-15

**Authors:** Paolo Perazzo, Riccardo Giorgino, Matteo Briguglio, Martina Zuffada, Riccardo Accetta, Laura Mangiavini, Giuseppe M. Peretti

**Affiliations:** ^1^Intensive Care Unit, IRCCS Orthopedic Institute Galeazzi, Milan, Italy; ^2^Orthopedics and Traumatology, University of Milan, Milan, Italy; ^3^IRCCS Orthopedic Institute Galeazzi, Milan, Italy; ^4^Traumatology Unit, IRCCS Orthopedic Institute Galeazzi, Milan, Italy; ^5^Regenerative and Reconstructive Unit, IRCCS Orthopedic Institute Galeazzi, Milan, Italy; ^6^Department of Biomedical Sciences for Health, University of Milan, Milan, Italy

**Keywords:** femoral fractures, anticoagulants, low-molecular-weight heparin, COVID-19 drug treatment, SARS-CoV-2

## Abstract

Proximal femoral fractures in older adults are not uncommon and represent a great challenge for orthopedic surgeons because of the high risks of complications. In the COVID-19 panorama, fractures occurring in infected older adults become an even more intricate task because of concomitant metabolic derangements due to SARS-CoV-2. Multidisciplinary protocols are mandatory and pharmacological treatment in infected patients should be tailored. Regrettably, the spread of the virus in northern Italy, has been faster than scientific progress in characterizing the disease and many hospitals have had to manage the symptoms on a daily clinical bases. Our Italian hospital in the region of Lombardy, which has been the epicenter of the Italian pandemic, has admitted sixteen patients with fractured femurs in March and April 2020. The first seven patients were treated with the antithrombotic prophylaxis of a single daily dose of low-molecular-weight heparin, but we observed the highest prevalence of deaths from cardiovascular complications (four deaths). By doubling the daily dose of anticoagulants in the subsequent patients, we observed a reduction in the incidence of death (one death out of nine). Controversies exist about the surgical treatment of fractures in older adults during this pandemic. However, we have observed an increased survival after fall trauma in infected older adults if treated with high doses of anticoagulant. Although not being statistically significant, our results are in line with the current knowledge of the pathophysiology of SARS-CoV-2 infection, but more studies should be shared about the efficacy and dosage of anticoagulants in traumatic injuries of the elderly.

## COVID-19 Pandemic: to Operate, or Not to Operate Fractures in Infected Older Adults?

Italy has been the worst-hit country in Europe during the pandemic of SARS-CoV-2 with 13.6% of mortality rates (WHO situation report 100 of April 29 2020). This disease named COVID-19 represents a severe health-care emergency, leading to a challenging management of trauma patients infected with the virus (1, 2). Italian hospitals have been involved in managing patients suffering from this infection and other comorbid conditions, with the health care system being rapidly saturated. At our orthopedic hospital in the region of Lombardy, which has been the epicenter of the Italian pandemic counting about 50% of all death in the country, has been reorganized to guarantee the best assistance to trauma emergencies (3). During this worldwide plague, the choice of operating older adults who have suffered a falling trauma has been a matter of debate. In the worst-hit countries, the experience of Chinese (4), Spanish (5), and colleagues from England (6) would suggest delaying the surgical treatment of fractured patients with SARS-CoV-2 or treating conservatively, as they have observed excessive mortality rates. The Italian experience would conversely suggest treating the fracture as soon as possible in order to stabilize the patient (7). Nevertheless, the choice depends on the type and severity of the fracture and is a fact that most of older adults that encounter a falling fracture face a life-treating event that need to be treated. For these patients, surgery has to be ensured within 48 h from admission (8) and supported with prophylactic anticoagulant therapy for venous thromboembolism consisting of a daily administration of low-molecular-weight heparin (LMWH, 4000 IU), which has been shown to decrease the risk of events by over 30% (9). The aim of this article is to share our anticoagulant management of fractured older adults infected with SARS-CoV-2 in order to discuss new criteria for the correct prevention of thrombotic events in COVID-19 patients. The ethical review and approval was not required for this case series study in accordance with the local legislation and institutional requirements.

## Methods in the Time of COVID-19

In March and April 2020, our orthopedic hospital counted sixteen emergency operations of non-deferrable femoral neck fractures. At admission, patients were tested for SARS-CoV-2 and categorized according to the 4-level classification of infected patients (therefore positive for the swab): level 0, asymptomatic, the patient should not be hospitalized; level 1, mild symptoms, pharyngodynia, dry cough, fever; level 2, moderate symptoms, high fever, persistent dry cough, asthenia, dyspnoea, requires oxygen support (non-invasive); level 3, severe symptoms, oxygen therapy (invasive), requires access to intensive care (10). Other than the collection of demographic data, clinical evaluations, and biochemical parameters, three classification indices were calculated comprising the CCI (Charlson Comorbidity Index) (11), the MUST (Malnutrition Universal Screening Tool) (12), and the ASA (American Society of Anesthesiologists). Patients received hydration, hydroxychloroquine treatment, and the anticoagulant therapy with LMWH. After surgery, all patients followed the early (within 48 h) physical exercises with physiotherapists since their functionality seemed not excessively altered by the concomitant infection.

## Results: From Standard-Dose to Escalated-Dose of Anticoagulant

**Figure 1:**
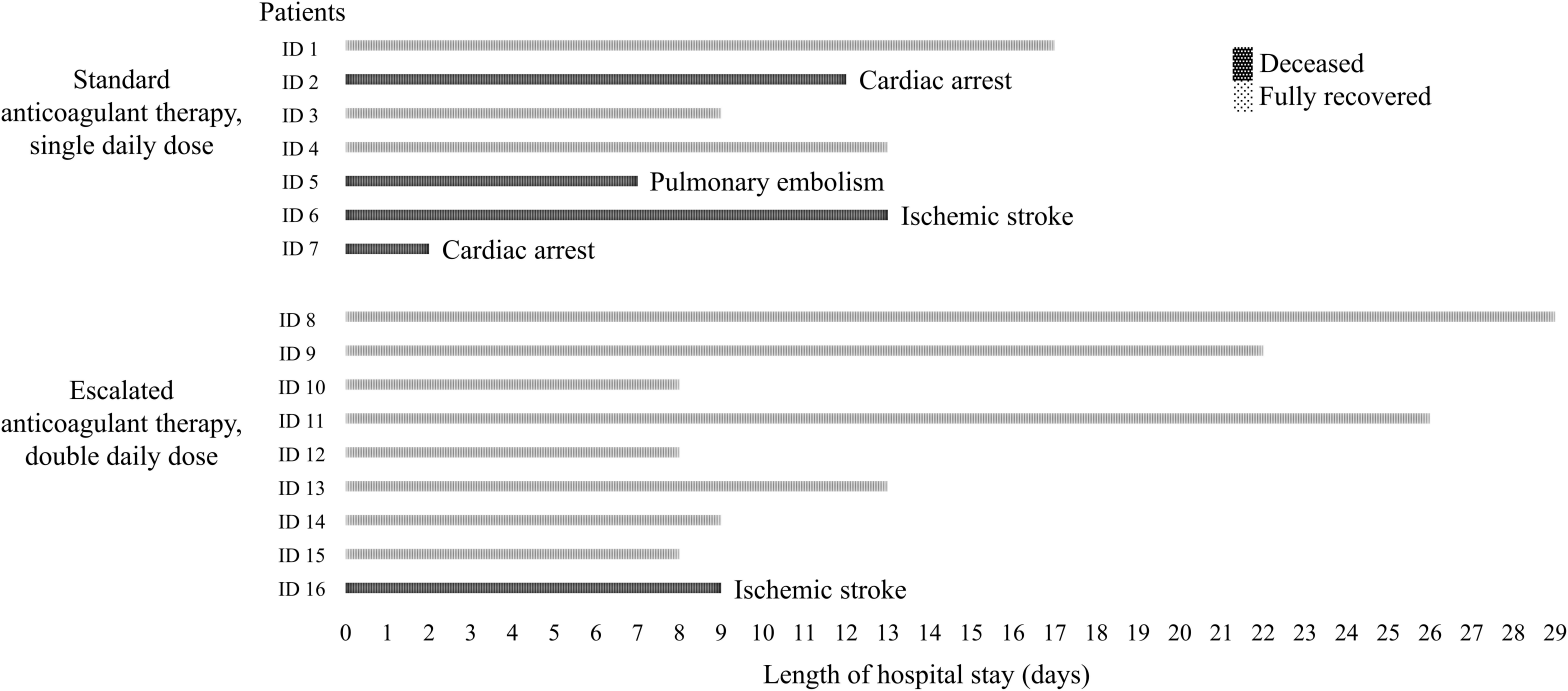
Survival outcome of older adult patients with SARS-CoV-2 after surgical treatment of femoral neck fracture and different anticoagulant therapy. During the months with the highest transmission and death rates for COVID-19 in Italy, sixteen older adults were admitted to an orthopedic hospital of northern Italy for a proximal femoral neck fracture. At admission, all patients resulted positive for SARS-CoV-2, with altered peripheral oxygen saturation, malnourished, mainly suffering from cardiovascular comorbidities, and with a level 2 severity for COVID-19 classification. The first consecutive seven patients were treated according to the standard anticoagulant prophylaxis, but four patients deceased for cardiovascular-associated causes. It was decided to double the dose of low-molecular-weight heparin for the subsequent patients and the death rate decreased from 57.1 to 11.1%.

At admission, the implemented access plan of the hospital assured an appropriate division of infected, probably infected, and non-infected subjects. At admission, the whole cohort counted two males and fourteen females with a mean age of 86.4 ± 6.2 years old all suffering of at least one cardiovascular condition (CCI = 4.4 ± 0.7), such as ischemic heart disease, hypertension, atrial fibrillation, or a combination of three. All patients resulted positive for SARS-CoV-2 and most of the subjects were disoriented in time and space rendering the proper assessment of COVID-19 symptoms hard to evaluate. The mild impairment of the peripheral oxygen saturation (all had over 90%) and the infective status allowed us to categorize the subjects as level 2 COVID-19 severity. All patients moderately suffered from limited activity (ASA score = 2.9 ± 0.4) and malnutrition (all were at high risk of malnutrition). Concerning the therapy with LMWH, the first seven patients were routinely supported with a standard prophylactic dose of 4000 IU. In particular, five patients received daily administration of Enoxaparin sodium and two patients received an equivalent prophylactic dosage of Nadroparin calcium based on the body weight. However, their clinical situation deteriorated rapidly within a few days and four patients had fatal cardiovascular events, being one ischemic stroke, two cardiac arrests in heart failure, and one pulmonary embolism. The observed cardiovascular mortality possibly associated with thrombotic events and the concomitant proposal of some scientific publications that suggested the prevalent hemostatic involvement in response to the virus were fundamental in deciding to double the dose of anticoagulant. In agreement with the hospital's health management, it has gone from standard-dose to escalated-dose of antithrombotic therapy. Therefore, the subsequent patients admitted for femoral neck fractures (nine consecutive patients) were treated with 4000 IU of Enoxaparin sodium twice a day. Still, one patient died for ischemic stroke. Overall, this second series was comparable to the first series for age (equal variances *T-*test: *p* = 0.107), malnutrition risk, comorbidity index, anesthesiology risk, and level of COVID-19 severity. No differences in the biochemical parameters at admission was found: hematocrit (equal variances *T-*test: *p* = 0.855), hemoglobin (equal variances *T-*test: *p* = 0.904), platelet count (equal variances *T-*test: *p* = 0.142), white blood-cell count (equal variances *T-*test: *p* = 0.732), C-reactive protein (equal variances *T-*test: *p* = 0.258), urea (equal variances *T-*test: *p* = 0.708), creatinine (equal variances *T-*test: *p* = 0.950), AST (equal variances *T-*test: *p* = 0.324), ALT (equal variances *T-*test: *p* = 0.180) (see Table 1 and Figure 1 for details). Despite not being statistically significant (2-sided Fisher's exact test: *p* = 0.106), the death rate decreased from 57.1 to 11.1%, which is a remarkable outcome different. Since autopsies were currently suspended for biohazard security concerns, all causes of death have been determined based on clinical evaluations. We found no statistical difference between survivors and non-survivors with regard to any routine biochemical parameter, which resulted to be rather normal for the conditions, or clinical symptom at admission. Although also the age between the single- and double-dose LMWH groups was not statistically different, the survivors had a mean of 84.6 ± 6.6 years vs. the 90.4 ± 2.7 years of non-survivors. By the end of April, eleven (68.8%) patients have been discharged and five (31.3%) patients have died.

**Table 1:** Data of older adults admitted to an Italian orthopedic hospital for femoral neck fracture in April 2020.

	**Standard anticoagulant therapy**,	**Escalated anticoagulant therapy**,
	**single daily dose of LMWH**	**double daily dose of LMWH**
**General demographics**		
Femoral fractured patients	Raw of 7	Raw of 9
Age (years)	89.3 ± 3.9 (82.0, 95.0)	84.2 ± 6.9 (72.0, 93.0)
Gender (ratio male:female)	Ratio 0:7	Ratio 2:7
Surgery period	First half of April 2020	Second half of April 2020
**Parameters at admission**		
SARS-CoV-2 quantitative RT-PCR	100% positive	100% positive
Peripheral oxygen saturation	100% over 90%	100% over 90%
Malnutrition Universal Screening Tool (MUST)	100% high risk	100% high risk
American Society of Anesthesiologists (ASA)	3.0 ± 0.6	2.9 ± 0.3
Charlson Comorbidity Index (CCI)	4.4 ± 0.8	4.4 ± 0.7
COVID-19 level of severity	100% level 2	100% level 2
Hematocrit (%)	35.6 ± 5.9 (29.8, 44.2)	36.2 ± 5.9 (27.8, 45.0)
Hemoglobin (g/dL)	11.9 ± 2.2 (9.6, 15.5)	11.7 ± 1.9 (8.9, 14.3)
Platelet count (10^3^/μL)	218.9 ± 85.2 (69.0, 316.0)	293.2 ± 101.4 (174.0, 469.0)
White blood-cell count (10^3^/μL)	10.4 ± 4.0 (4.3, 15.6)	11.2 ± 5.5 (4.0, 23.1)
C-reactive protein	2.5 ± 3.6 (0.1, 8.7)	4.6 ± 3.7 (0.0, 10.3)
Urea (mg/dL)	48.6 ± 20.2 (28.0, 83.0)	53.8 ± 31.1 (19.0, 120.0)
Creatinine (mg/dL)	0.8 ± 0.3 (0.5, 1.4)	0.8 ± 0.4 (0.3, 1.5)
AST (U/L)	32.4 ± 15.2 (18.0, 60.0)	25.3 ± 12.6 (14.0, 49.0)
ALT (U/L)	22.3 ± 12.9 (8.0, 45.0)	15.7 ± 5.2 (7.0, 23.0)
**Clinical outcome**		
Length of hospital stay	10.4 ± 4.9	14.7 ± 8.6
Discharged with recovery	3	8
Deceased	4	1

*CCI scores 1–2 = mild. CCI scores 3–4 = moderate. CCI scores ≥ 5 = severe*.

*COVID-19 level of severity: classification of the infected patient (therefore positive for the swab) according to 4 levels at admission. Level 0: asymptomatic, the patient should not be hospitalized. Level 1: mild symptoms, pharyngodynia, dry cough, fever. Level 2: moderate symptoms, high fever, persistent dry cough, asthenia, dyspnoea, requires oxygen support (non-invasive). Level 3: severe symptoms, oxygen therapy (invasive), requires access to intensive care*.

## Discussion

We here compared the survival in two consecutive series of patients, among them overlapping for clinical conditions and hospital path, which have undergone two different perioperative dosages of LMWH for proximal femoral neck fracture in March-April 2020. Notably, all patients resulted to be at high risk of malnutrition, being the alteration of the nutritional status a predisposing factor both for SARS-CoV-2 infection (13) and traumatic falls (14). Despite the slight similarity, the survivors were younger than those deceased and it is known that the older the age the higher the case fatality in individuals suffering from COVID-19 (15). The low platelet count is also known to be associated with increased mortality in patients with COVID-19 (16), but we found no differences between the group of survivors and deceased (260.0 ± 79.3 10^3^/μL and 262.2 ± 145.4 10^3^/μL, respectively). Of note, the patient that died of cardiac arrest in the first series of patients had thrombocytopenia of 69.0 10^3^/μL and accounted for the high standard deviation of the group. Our results show that the use of higher doses of LMWH may be associated with improved survival in older adults with COVID-19 after surgical treatment for fracture, but the lack of biochemical monitoring does not allow to conclude that the tendency to reduce mortality is due to a higher dose of anticoagulant. Notably, the number of transfusions in the escalated heparin group was more than double that in the standard dose group (18 vs. 8), definitely reflecting the drug-depended prolongation of the time that blood takes to clot. To the best of our knowledge, this is the first report in Orthopedics discussing the possible advantages on survival of higher doses of LMWH compared to standard prophylaxis. Despite our observation cannot be generalized beyond the context of patients' cases, it appears reasonable that an escalated dose of anticoagulant would effectively protect the surgical patient from venous thromboembolism associate with SARS-CoV-2 infection. In this regard, more attention should be paid on the associated bleeding risk. Many severe COVID-19 patients were reported to meet the criteria for the disseminated intravascular coagulation (consumptive of both platelets and clotting factors) (17). In these patients, clot formation was associated with spleen atrophy, hilar lymph node necrosis, and hepatomegaly (18). Whatever is the mechanism underlying the infection-associated thrombosis (that is likely to be multifactorial), the coagulopathy observed in COVID-19 patients is likely to follow the “two-activation theory of the endothelium” (19), with both the release of inflammatory cytokines and the activation of platelets that trigger the activation of inflammatory and microthrombotic pathways. This cascade of events has been observed in severe influenza pneumonia (20), but it is also known to be the underlying pathogenesis for ischemic heart diseases (acute coronary syndrome), stroke, and venous thromboembolism (which includes deep vein thrombosis and pulmonary embolism).

Current guidelines from the National Institutes of Health state that there is still insufficient data to recommend either for or against using escalated or therapeutic doses of antithrombotic agents in infected patients, and still the evidences are based on expert opinions (21–23). The clinical efficacy of heparin in decreasing mortality rates in COVID-19 has been suggested by colleagues from China and United States of America (24–26), but they did not discuss the dose per patient though. It should be considered that most of the evidences on the use of anticoagulants has not been based on trauma older adults. Notably, these patients normally face an already very high risk of basal thromboembolic complications and we therefore believe that surgery with a double dose of anti-thromboembolic prophylaxis represents the most appropriate treatment for these patients with femoral fractures. Although our results should not be used to support changes in clinical practice because of the observational nature of the study and the lack of statistical significance, older adults receiving a standard dose of anticoagulant were more at risk of death than those receiving a double dose (relative risk 5.14, CI 95% 0.73:36.37). In the near future, we are looking forward to seeing results from large prospective randomized trials that investigate whether higher doses of LMWH would protect against thrombotic events in COVID-19 patients undergoing surgery. Monitoring of coagulation parameters and bleeding events should be included. In addition, it would be interesting to focus research on the potential antiviral role of heparin (27).

## Data Availability Statement

The datasets generated for this study can be found in online repositories. The names of the repository/repositories and accession number(s) can be found in the article/Supplementary Material.

## Ethics Statement

Ethical review and approval was not required for the study on human participants in accordance with the local legislation and institutional requirements. Written informed consent for participation was not required for this study in accordance with the national legislation and the institutional requirements.

## Author Contributions

PP formulated the hypothesis. RG and MB wrote the first draft of the manuscript. MZ, RA, LM, GP, and PP revised the first draft and contributed to manuscript sections. All authors contributed to manuscript revision, read, and approved the submitted version.

## Conflict of Interest

The authors declare that the research was conducted in the absence of any commercial or financial relationships that could be construed as a potential conflict of interest.
